# Engineering Bifunctional Calcium Alendronate Gene‐Delivery Nanoneedle for Synergistic Chemo/Immuno‐Therapy Against HER2 Positive Ovarian Cancer

**DOI:** 10.1002/advs.202204654

**Published:** 2023-03-18

**Authors:** Guochuang Chen, Leli Zeng, Bo Bi, Xiuyu Huang, Miaojuan Qiu, Ping Chen, Zhi‐Ying Chen, Yulong He, Yihang Pan, Yu Chen, Jing Zhao

**Affiliations:** ^1^ Syno Minicircle Biotechnology Shenzhen 518055 P. R. China; ^2^ Precision Medicine Center Scientific Research Center The Seventh Affiliated Hospital of Sun Yat‐sen University Shenzhen 518107 P. R. China; ^3^ Materdicine Lab School of Life Sciences Shanghai University Shanghai 200444 P. R. China

**Keywords:** alendronate, bispecific T‐cell engager, gene delivery, immunotherapy, ovarian cancer

## Abstract

Ovarian cancer is the most lethal gynecological malignancy. Most patients are diagnosed at an advanced stage with widespread peritoneal dissemination and ascites. Bispecific T‐cell engagers (BiTEs) have demonstrated impressive antitumor efficacy in hematological malignancies, but the clinical potency is limited by their short half‐life, inconvenient continuous intravenous infusion, and severe toxicity at relevant therapeutic levels in solid tumors. To address these critical issues, the design and engineering of alendronate calcium (CaALN) based gene‐delivery system is reported to express therapeutic level of BiTE (HER2×CD3) for efficient ovarian cancer immunotherapy. Controllable construction of CaALN nanosphere and nanoneedle is achieved by the simple and green coordination reactions that the distinct nanoneedle‐like alendronate calcium (CaALN‐N) with a high aspect ratio enabled efficient gene delivery to the peritoneum without system in vivo toxicity. Especially, CaALN‐N induced apoptosis of SKOV3‐luc cell via down‐regulation of HER2 signaling pathway and synergized with HER2×CD3 to generate high antitumor response. In vivo administration of CaALN‐N/minicircle DNA encoding HER2×CD3 (MC‐HER2×CD3) produces sustained therapeutic levels of BiTE and suppresses tumor growth in a human ovarian cancer xenograft model. Collectively, the engineered alendronate calcium nanoneedle represents a bifunctional gene delivery platform for the efficient and synergistic treatment of ovarian cancer.

## Graphical abstract

Alendronate calcium‐based physical perforation gene delivery technique is designed to realize synergistic chemo/immuno‐therapy against HER2 positive ovarian cancer. Alendronate calcium not only induces apoptosis of SKOV3 cell via down‐regulation of HER2 signaling pathway, but also mediates expression of therapeutic levels of BiTE and synergizes with BiTE (HER2×CD3) to generate high antitumor response.

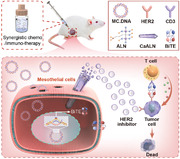

## 1 Introduction

For decades, there has been a dramatic expansion of the innovative therapies for patients with ovarian cancer, including vascular endothelial growth factor receptor (VEGF) inhibitors and poly(adenosine 5'‐diphosphate [ADP]‐ribose) polymerase (PARP) inhibitors.^[^ ^1^, ^2^ ^]^ Despite these advances, ovarian cancer is still the most lethal of the gynecologic malignancies. Thus, there is a substantial need to develop therapeutic interventions with novel mechanisms of action, particularly for patients with advanced or resistant ovarian cancer.

HER2‐targeted therapies, including antibodies (trastuzumab and pertuzumab), antibody‐drug conjugate (ADC), and tyrosine kinase inhibitor have significantly improved patient outcomes in breast and gastric cancer.^[^ ^3^ ^]^ However, their applications in ovarian cancer are debatable and clinical evidences have demonstrated that patients with HER2‐positive ovarian cancer showed poor response to existing HER2‐targeted therapy.^[^ ^4^, ^5^ ^]^ Recently, immunotherapy has been developed to combat advanced‐stage cancers in clinic. As the key component of immune system, cytotoxic T lymphocytes (CTLs) can recognize and kill aberrant and virally infected cells by secreting cytolytic proteins, including perforin and granzyme B.^[^ ^6^ ^]^ The patients maybe conceivably benefit from some forms of immunotherapy, including bispecific antibody (BsAb)‐based T cells engaging therapy and engineered T cells‐based adoptive therapy.^[^ ^7^ ^]^

Bispecific antibodies are engineered artificial antibodies capable of simultaneously recognizing two or more epitopes, and the formats with dual binding site for tumor‐associated antigen (TAA) and CD3 on T cells are referred to T cell‐engagers or T cell‐engaging BsAbs. T cell‐engagers bridge the proximal contact of T cells and cancer cells, and prompt the formation of cytotoxic synapses triggering tumor specific response.^[^ ^8^ ^]^ A minimalistic T cell‐engager is described as a tandem single‐chain Fv (scFv) fusion protein binding to tumor‐associated antigen and CD3 on T cells, which is connected through a flexible linker.^[^ ^9^ ^]^ This format presents the classical basis of bispecific T‐cell engager (BiTE) molecules. The properties of BiTEs have been meticulously studied and they have emerged as a new pillar of cancer treatment. Immunotherapy of cancer with CD3‐engaged BiTE format has been approved for some hematological malignancies and extensively applied in the clinical investigation for solid cancers.^[^ ^10^ ^]^ These entities possess compact configuration and lack Fc region, with a molecular mass in the range of 50–60 kDa, which could be advantageous regarding tumor tissue penetration and distribution. Despite potent efficiency, inconvenience, and high cost of treatment hampered its broad application. The half‐life of this format is less than a few hours, which is unavailable for intermittent administration in patients.

A promising and alternative strategy is in vivo delivery of synthetic nucleic acid ectopically or in situ, with high potential to circumvent the critical challenges.^[^ ^11^ ^]^ Iwahori and colleagues engineered T cells to secrete EphA2×CD3 BiTE using retroviral vectors.^[^ ^12^ ^]^ However, the risks of retroviral integration in the genome and immunogenicity prompt the development of nonviral gene transfer methods, including nanoparticle delivery and physical technology. RNAs and DNA encoding BiTEs were systemically transferred in vivo by polymer or lipid‐based transfection reagent or locoregionally delivered into specific cells via electroporation, even into muscle by the simple direct naked DNA injection.^[^ ^13^, ^14^, ^15^ ^]^ To mitigate adverse toxicity associated with systemic exposure to BiTEs and enhance antitumor efficiency, we focus on the development of efficient locoregional gene delivery approaches.

Nitrogen‐containing bisphosphonates (NBPs), mainly including alendronate and zoledronic acid, are potent inhibitors of osteoclast‐mediated bone resorption and are commonly applied for osteoporosis and skeletal metastases. It was extensively reported that these molecules could block tumor progression by immunomodulatory effects, direct proapoptotic activity, and reducing adhesion, migration, and invasion.^[^ ^16^, ^17^, ^18^, ^19^ ^]^ Intriguingly, one of the underlying molecular mechanisms of NBPs action is global down‐regulation of human epidermal growth factor receptors (Her) signaling pathway by blocking kinase domain.^[^ ^20^ ^]^ In the *Her‐2/neu* transgenic primary tumor mouse model, bisphosphonates switched immunosuppression in a harsh tumor microenvironment and inhibited spontaneous mammary cancerogenesis.^[^ ^21^ ^]^ HER2 amplification or overexpression occurred in ovarian cancer ranging from 5% to 30%.^[^ ^22^, ^23^ ^]^ The subpopulation of ovarian cancer patients with high HER2 expression levels could conceivably benefit from the combination of NBPs and HER2‐targeted T‐cell engaging immunotherapy.

**Figure 1:**
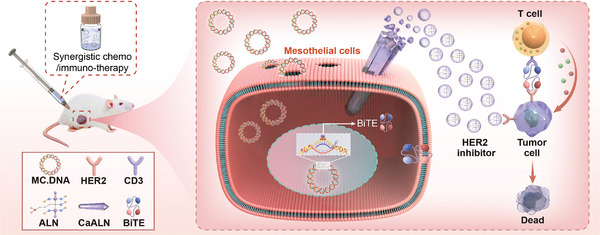
The design of bifunctional calcium alendronate gene‐delivery nanoneedle for synergistic chemo/immuno‐therapy against HER2 positive ovarian cancer.

Herein, a minicircle DNA (MC) encoding a BiTE format targeted HER2 and CD3 (HER2×CD3) was designed for HER2 positive ovarian cancer immunotherapy. The nanosized alendronate calcium (CaALN), especially those 1D nanoneedle (CaALN‐N) served as a highly efficient gene delivery system via the intraperitoneal (IP) administration route. The gene delivery and anticancer effects of CaALN‐N were evaluated in vitro and in tumor‐bearing animal models. CaALN‐N mediated durable expression of MC‐HER2×CD3, and featured the potential to promote polyclone T cell activation and retard tumor progression of human ovarian cancer intraperitoneal xenograft (**Figure** **1**). Overall, this engineered CaALN nanoneedle with the advantages of chemotherapeutic and gene transfection activities represents a powerful tool for abdominal tumors.

## 2 Results and Discussion

### 2.1 Design, Synthesis, and Characterization of CaALN Nanoneedle and Nanosphere

**Figure 2:**
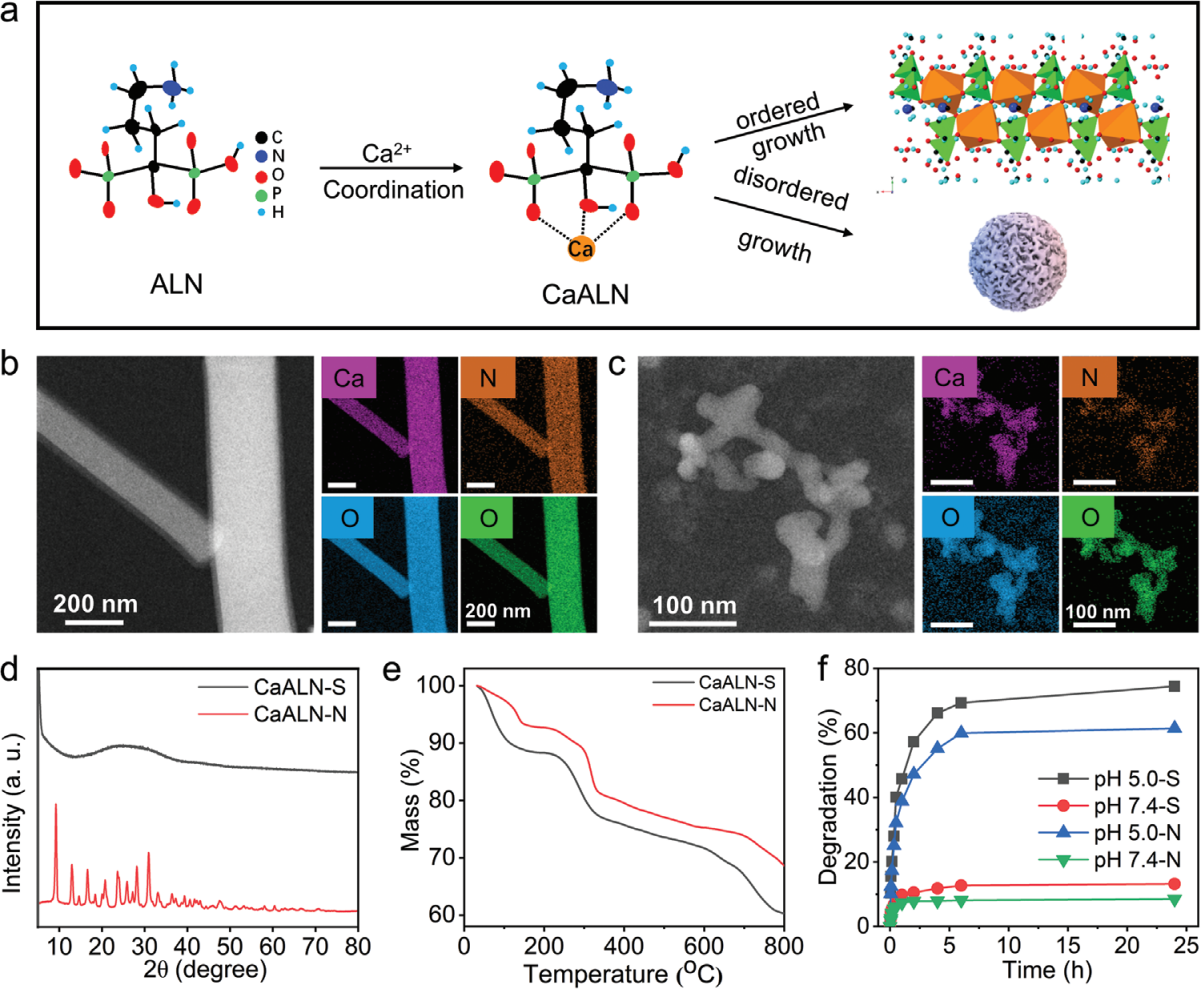
Controllable preparation and characterization of CaALN. a) Schematic diagram of growth of CaALN nanosphere and nanoneedle. b) TEM image and corresponding mapping chemical elements of CaALN‐N. c) TEM image and corresponding mapping chemical elements of CaALN‐S. d) XRD patterns of CaALN‐N and CaALN‐S. e) TG curves of CaALN nanosphere and nanoneedle. f) The degradation of CaALN‐N and CaALN‐S in phosphate‐buffered saline (PBS) with pH of 5.0 or 7.4.

Alendronate, a clinically approved drug for the treatment of osteolytic metastases, has a backbone structure of P‐C‐P where P is a phosphonate group. As shown in **Figure** **2**a, phosphate groups of alendronate can coordinate with Ca^2+^ in the solution. The growth of the CaALN cluster could be controlled by the change in feeding ratio, reaction time, and temperature. As shown in Figure S1, Supporting Information, pure CaALN crystal (nanoneedle, CaALN‐N) could be obtained by reaction of alendronate and Ca^2+^ at a molar ratio of 1.5, pH 5.0, and heating at 60 and 90 °C. As demonstrated in the XRD pattern of crystalline CaALN (bottom of Figure 2d), all peaks were well indexed as orthorhombic a Ca[C_4_H_11_NO_7_P_2_]•H_2_O (space group P2_1_2_1_2_1_). TEM and SEM images revealed that the CaALN nanoneedle was about 5 µm in length and 200 nm in width (Figure 2b; and Figure S2a, Supporting Information). Amorphous CaALN nanospheres (CaALN‐S) were obtained at the relatively low temperature and high pH value. The CaALN nanospheres exhibited rod‐like morphology with the average size of 50 nm (Figure 2c; and Figure S2b, Supporting Information). The mass loss of thermo gravimetry (TG) curves before 100 °C showed that amorphous CaALN nanospheres had more water in their structures than crystalline CaALN (Figure 2e). The remaining mass loss was attributed to the decomposition of organic molecules in air atmosphere. The degradation of CaALN were detected by incubating CaALN powders in phosphate‐buffered saline (PBS) with pH of 5.0 or 7.0, indicating that the degradation rate of amorphous CaALN nanospheres was higher than crystalline CaALN nanoneedle in acidic environment (pH = 5.0), while the degradation of both of them were less than 15% in neutral condition (pH = 7.0) (Figure 2f).

### 2.2 Determination of CaALN Mediated Gene Delivery In Vivo

**Figure 3:**
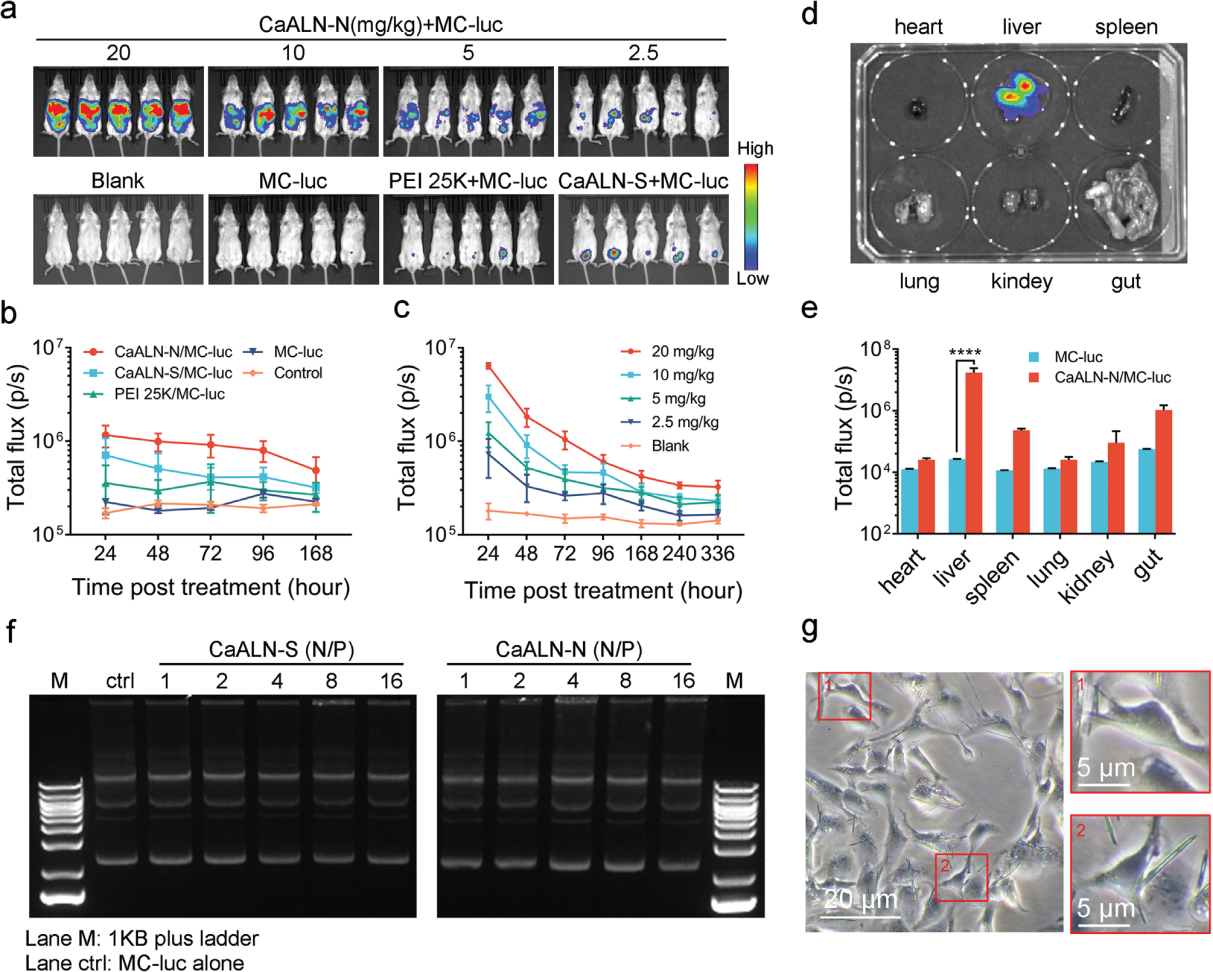
Determination of CaALN mediated delivery efficiency in vivo. BALB/c mice were IP injected with CaALN‐N and MC‐luc in 400 µL PBS buffer or PEI 25 K and the expression of lucifease was monitored by IVIS. a) Representative IVIS images of groups 12 h post treatment. b) The transfection efficiency was quantified as the luciferase activity signal (total flux) from BLI imaging at the indicated time point (*n* = 5, mean ± SD). c) Quantitation of luciferase activity mediated by a serial dose of CaALN‐N (*n* = 5, mean ± SD). d) The animals in CaALN‐N at 5 mg kg^−1^ group were autopsied and analyzed by IVIS for detailed organ‐specific distribution of luciferase positive cells (*n* = 5). e) The luciferase expression in each organ was quantified as total flux (photons/s) from IVIS imaging of each mouse (*n* = 5, mean ± SD, ****p < 0.0001). f) CaALN and MC‐luc were mixed and incubated for 30 min and separated on 1% agarose gel. g) Co‐incubation of SKOV3‐luc cells with CaALN for 24 h and photographed by confocal microscopy.

Nanostructures with high aspect ratios, including nanoneedles, nanowires (NWs), nanoflowers, and nanostraws, represent physical means to transiently permeabilize the cell membrane and enable intracellular delivery of biomacromolecules both in vitro and in vivo.^[^ ^24^, ^25^ ^]^ We next tested whether CaALN crystal and nanospheres could serve as gene delivery system. MC encoding luciferase (MC‐luc) was used as reporter gene to evaluate the gene delivery efficiency of CaALN. To prepare the transfection system, equal volume of MC‐luc at 0.1 mg mL^−1^ was mixed with CaALN at 1.0 mg mL^−1^ in Ca^2+^/Mg^2+^ free PBS. 400 µL of the mixture was IP injected into BALB/c mice. A commercial gene transfection reagent PEI 25k was used as control group. The bioluminescent signal of luciferase reporter gene was captured by the IVIS spectrum imaging system. Mice treated with CaALN‐N/MC‐luc displayed intense bioluminescence on the abdomen, especially the top region, and the signal was detectable for 5 days (**Figure** **3**a,b; and Figure S3, Supporting Information). However, PEI 25k treated mouse exhibited a weaker bioluminescence signal. Next, serially titrated doses of CaALN‐N with MC‐luc were IP injected in the mice and the bioluminescence data were shown in Figure 3c. As expected, higher transfection efficiency was achieved following dose escalation of CaALN‐N and sustained expression of luciferase for at least 14 days (Figure S4, Supporting Information). In addition, the CaALN‐N mediated gene transfection in nude mice was confirmed, which showed similar expression pattern with BALB/c mice (Figure S5, Supporting Information). Besides, several mice showed occasional bioluminescence signals in the lower abdomen across PEI 25K/MC‐luc, CaALN‐S/MC‐luc, and CaALN‐N/MC‐luc groups. The bioluminescence signals in the lower abdomen were located at the puncture point of syringe. In previous research, intramuscular injection of MC‐luc or plasmid was able to persistently express transgene product.^[^ ^13^, ^26^, ^27^ ^]^ We speculated that PEI and CaALN‐S/N stimulated the puncture site resulting in little leak of MC‐luc into muscle of abdominal wall. Thus, we explored the distribution of CaALN‐N in vivo. As shown in Figure S6, Supporting Information, the fluorescence signal of Cy7 conjugated CaALN‐N did not overlap with SKOV3‐luc xenograft in nude mice, which indicated that CaALN‐N had no obvious tumor targeting capacity. It seemed that CaALN‐N could widely distribute in the whole abdominal cavity. Some CaALN‐N retained around injection site and could irritate the puncture injury resulting in leak of little MC‐luc into abdominal muscle, which could be an explanation for the occasional bioluminescent spot in the lower abdomen of mice.

These results prompted us to explore the underlying mechanisms of CaALN‐N mediated transfection. We performed an extensive postmortem analysis of treated mice and collected their visceral organs for bioluminescent imaging. It was found that liver showed apparent stronger bioluminescence signal in comparison with other organs (Figure 3d,e). Using gel electrophoresis, we found that the presence of CaALN‐S or CaALN‐N did not specifically change the prominent bands of MC‐luc, which revealed no marked interaction among CaALN‐N and MC‐luc (Figure 3f). CaALN‐N preserved its structural integrity in simulated physiological conditions for more than 24 h (Figure 3g). Interestingly, CaALN‐N penetrated in adherent SKOV3‐luc cells was captured by confocal microscopy (Figure S7, Supporting Information), which indicated that CaALN‐N physically interacted with cytomembrane and created reversible holes for the transmembrane translocation of transgene. Liver and gastrointestinal tracts were the major organs exposed to CaALN‐N and MC‐luc. The liver was smooth and maintained tissue tension and pressure.^[^ ^28^ ^]^ Thus, The external surface of liver was susceptible to puncture. By comparison, the existence of wrinkled structure and soft‐elastic property of gastrointestinal tracts exhibited remarkable resistance to the puncture injury.^[^ ^29^, ^30^ ^]^ Upon gastrointestinal, diaphragm, and somatic motility or massage‐like stroking of the abdomen, relative motion between the peritoneum and its surrounding structure occurred. In IP injection route, CaALN‐N/MC‐luc solution was concentrated and evenly dispersed on the surface of abdominal viscera. The sharp tip of CaALN‐N mechanically breached the cell membrane of the visceral peritoneum enclosed liver tissue and allowed transgenes to diffuse across the permeabilized cell membrane (Figure 1).

### 2.3 CaALN‐N Exhibited Higher Cytotoxicity to Human Ovarian Cancer Cells than Normal Cells

**Figure 4:**
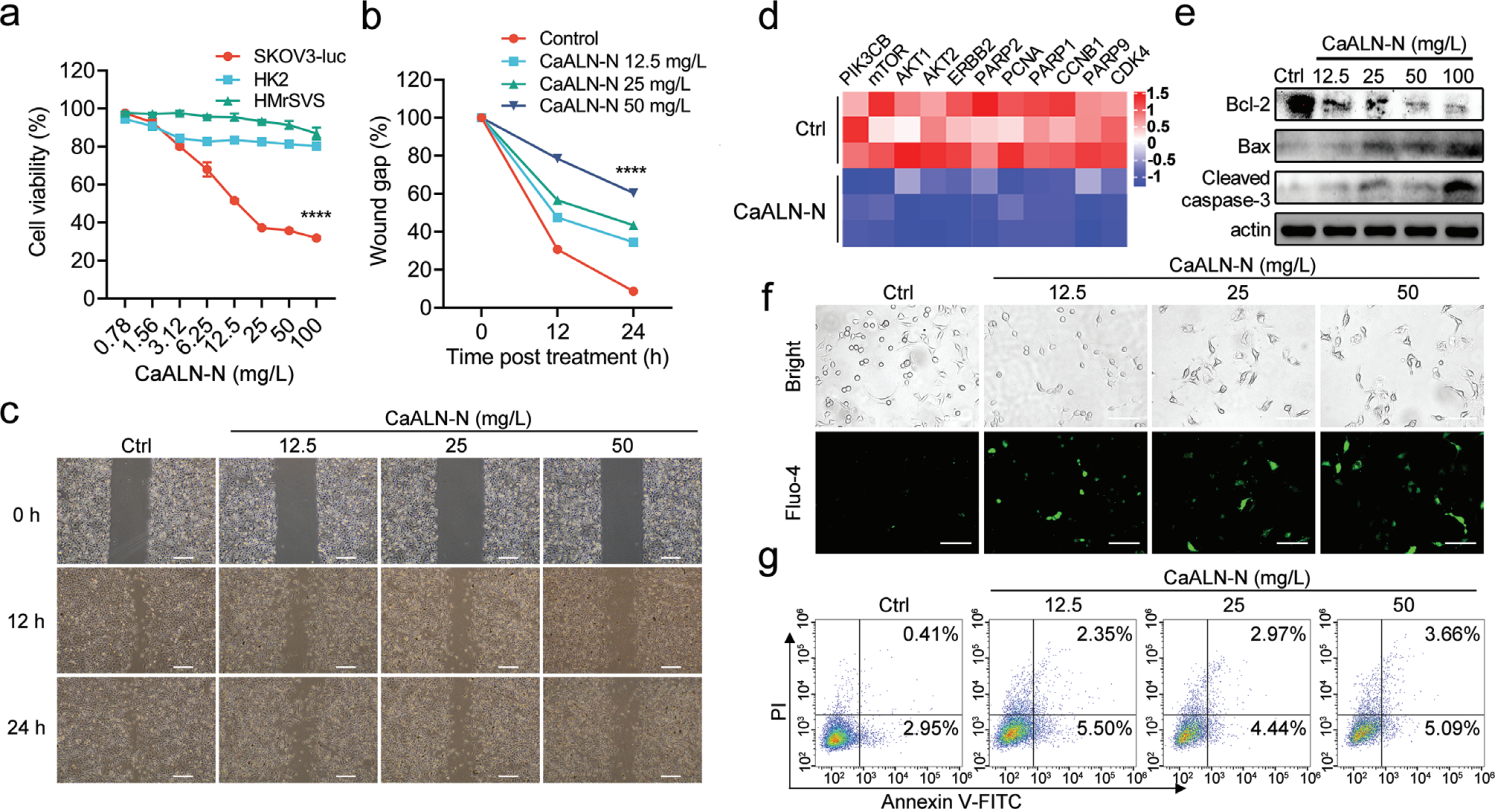
CaALN‐N displayed direct cytotoxicity on human ovarian cancer cells. a) SKOV3‐luc cells, HK2 and HMrSV5 cells were incubated with CaALN‐N for 16 h. The cell viability was detected by CCK8 kit or bright‐glo luciferase assay system (*n* = 3, mean ± SD). ****p < 0.0001 (compared to control group). b) The distance of each scratch in the monolayer was quantified and calculated relative to 0  h. Data are presented as mean ± SD. c) Images were captured at 0, 12, and 24 h, and representative images were shown. The scale bar was 250 µm. d) Heatmap illustrated the relative expression of the 11 differentially expressed HER2 pathway genes in SKOV3‐luc cells treated by CaALN‐N for 24 h compared to control group (*n* = 3 biological replicates). e) The expression of apoptotic pathway associated proteins in SKOV3‐luc cells treated with CaALN‐N was analyzed by western‐blot. SKOV3‐luc cells were treated with indicated concentrations of CaALN‐N for 16 h, followed by f) Fluo‐4/AM probe for confocal microscopy with scale bar 100 µm and g) flow cytometry assay with Annexin V‐FITC/PI staining.

HER2 is a member of the human epidermal growth factor receptor family of transmembrane tyrosine kinase receptors that are important mediators of cell growth, development, and survival.^[^ ^31^ ^]^ HER2 expression is frequently amplified and/or overexpressed and associated with poor prognosis in ovarian cancer.^[^ ^32^, ^33^ ^]^ There is a rapidly growing number of evidence highlighted the potential of novel HER2‐targeted therapy. In physiological conditions, slow degradation of CaALN‐N was conformed. Thus, we next investigated whether CaALN‐N could generate antitumor effects. As shown in **Figure** **4**a–c, CaALN‐N impeded the proliferation and migration of SKOV3‐luc cells. These results were coincident with the observation that less cytotoxicity was detected in HER2 negative normal cells HK2 and HMrSV5 (Figure 4a). Western blots on whole cell extracts confirmed overexpression of HER2 in human ovarian cancer SKOV3‐luc cells, but not in normal cells (Figure S8, Supporting Information). Several reports demonstrated that inactivation of human epidermal growth factor receptors and mevalonate‐Rho signaling axis resulted in apoptosis and inhibition of migration in human ovarian cancer.^[^ ^20^, ^34^ ^]^ The downstream targets of HER2 signaling cascade related to cell cycle, DNA repair and cell proliferation. We performed transcriptome sequencing to identify the status of HER2 signaling pathway in CaALN‐N treated SKOV3‐luc cells. As shown in Figure 4d, HER2‐PI3K‐AKT‐mTOR axis is dramatically down‐regulated by CaALN‐N treatment. In addition, CaALN‐N markedly down‐regulated the expression of Bcl‐2, accompanied by increase of Bax and cleaved caspase‐3 in SKOV3‐luc cells (Figure 4e). Consistently, calcium homeostasis imbalance and apoptosis were observed in SKOV3‐luc cells treated by CaALN‐N (Figure 4f,g). Collectively, these data showed that the inhibition of HER2 signaling pathway and triggering apoptosis accounted for the potent antitumor activity of CaALN‐N in HER2 over‐expression cancer cell line.

### 2.4 HER2×CD3 Induced T Cell‐Mediated Cytotoxicity Against HER2+ Human Ovarian Cancer Cells

**Figure 5:**
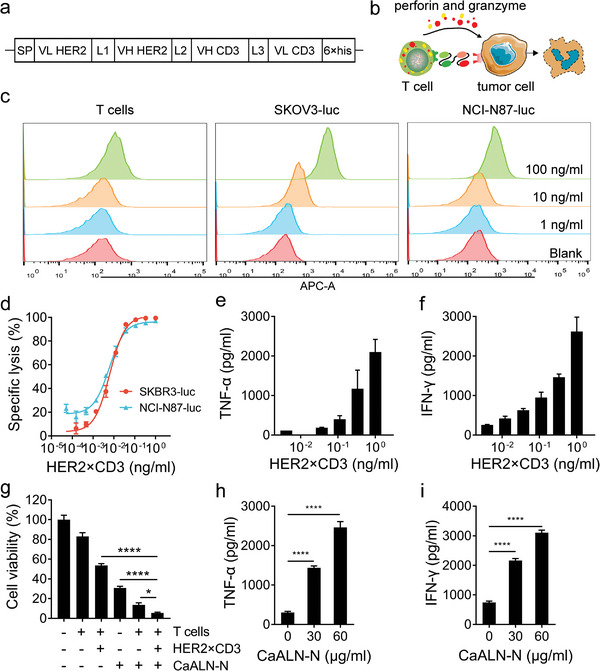
HER2×CD3 targeted HER2 to activate T cells in vitro. a) Schematic illustration of the structure of HER2×CD3 bispecific antibody. HER2×CD3 was composed of scFv to HER2 and scFv to CD3*ε*. L1, linker 1 (G4S1)3; L2, linker 2 G4S1; L3, linker 3 GEGTSTGSGGSGGSGGAD; VL, light chain variable region; VH, heavy chain variable region; 6×his, a tag for convenient purification of the bispecific antibody. b) Schematic illustration of action of HER2×CD3. The red and yellow dots were granzyme and perforin, respectively. c) Binding of HER2×CD3 to HER2 positive cancer cells (ovarian cancer SKOV3‐luc and gastric cancer) and CD3 positive T cells. d) HER2 positive cancer cells and T cells were co‐cultured with serial dilutions of HER2×CD3 for 14 h. The special lysis was assayed by bright‐glo luciferase assay system. e) TNF‐*α* and f) IFN‐*γ* in the supernatant were detected, respectively. g) The SKOV3‐luc cells and T cells were incubated with HER2×CD3 in the presence or absence of CaALN‐N for 16 h, followed by bright‐glo luciferase assay. The supernatants were analyzed for h) TNF‐*α* and i) IFN‐*γ* (*n* = 3, mean ± SD). *p < 0.05, ****p < 0.0001.

HER2×CD3 mediated T‐cell killing activity is independent of HER2 signaling pathway, but dependent on HER2 expression on tumor cell, thereby overcoming resistance to therapeutic monoclonal antibodies, and targeted small‐molecule inhibitors. We constructed a MC vector containing a BiTE‐targeting human HER2 and CD3 (HER2×CD3) expression cassette (**Figure** **5**a). HER2×CD3 consisted of two scFvs (anti‐HER2 from trastuzumab and anti‐CD3 from clone diL2K) connected through a G4S linker in the VL_HER2_‐VH_HER2_‐VH_CD3_‐VL_CD3_ orientation. As expected, HER2×CD3 could simultaneously targeted T cells and HER2‐positive cancer cells, which resulted in immunological synapse formation and effective T cell‐mediated cytotoxicity (Figure 5b).

First, we produced purified HER2×CD3 using shaking culture of transiently transfected 293 cells and his‐tag purification resin. And the purity and molecular weight of HER2×CD3 were confirmed by sodium dodecyl sulfate polyacrylamide gel electrophoresis under reducing and nonreducing conditions (Figure S9, Supporting Information). The binding capacity of HER2×CD3 on endogenously expressing cells was determined by flow cytometry assay. As shown in Figure 5c, HER2×CD3 had the expected binding specificity to CD3+ T cells and HER2+ cancer cells (human ovarian cancer SKOV3‐luc and human gastric cancer NCI‐N87‐luc). We next tested whether T cells could be activated by HER2×CD3 to kill HER2 positive cancer cells. When HER2×CD3 was applied to coculture HER2+ tumor cells and CD3+ T cells, a dose‐dependent cytotoxicity and cytokines release, including tumor necrosis factor (TNF)‐*α* and interferon (IFN)‐*γ*, were observed (Figure 5d–f). The HER2×CD3 mediated cytotoxicity of T cells against SKOV3‐luc cells was further confirmed by propidium iodide (PI) staining, and dead cancer cells emitted bright red fluorescence (Figure S10, Supporting Information). In the absence of T cells, HER2×CD3 showed no direct toxicity on SKOV3‐luc cells (Figure S11, Supporting Information).

The HER2/PI3K/AKT pathway plays a causal role in the drug resistance of ovarian cancer cells.^[^ ^35^, ^36^ ^]^ HER2 inhibitors have been shown to sensitize cancer cells and facilitated antitumor effects of immunotherapy.^[^ ^37^, ^38^ ^]^ Since CaALN‐N generated potent antitumor effects by global reduction of cell survival pathway, cell‐cycle arrest, and apoptosis, we next investigated whether it can sensitize SKOV3‐luc cells to the tumoricidal activity of HER2×CD3 armed T cells. As shown in Figure 5g, combination of CaALN‐N and HER2×CD3 armed T cells induced more effective antitumor effects than that alone. The proinflammatory cytokines, including TNF‐*α* and IFN‐*γ*, were also more potently induced in the presence of CaALN‐N (Figure 5h,i). Thus, these results confirmed the synergistic effects of CaALN‐N and BiTE‐engaged T cells, which revealed a potential therapeutic strategy against cancer.

### 2.5 CaALN‐N/MC‐HER2×CD3 Generated Potent Antitumor Effects in Human Ovarian Cancer Intraperitoneal Xenograft Model

**Figure 6:**
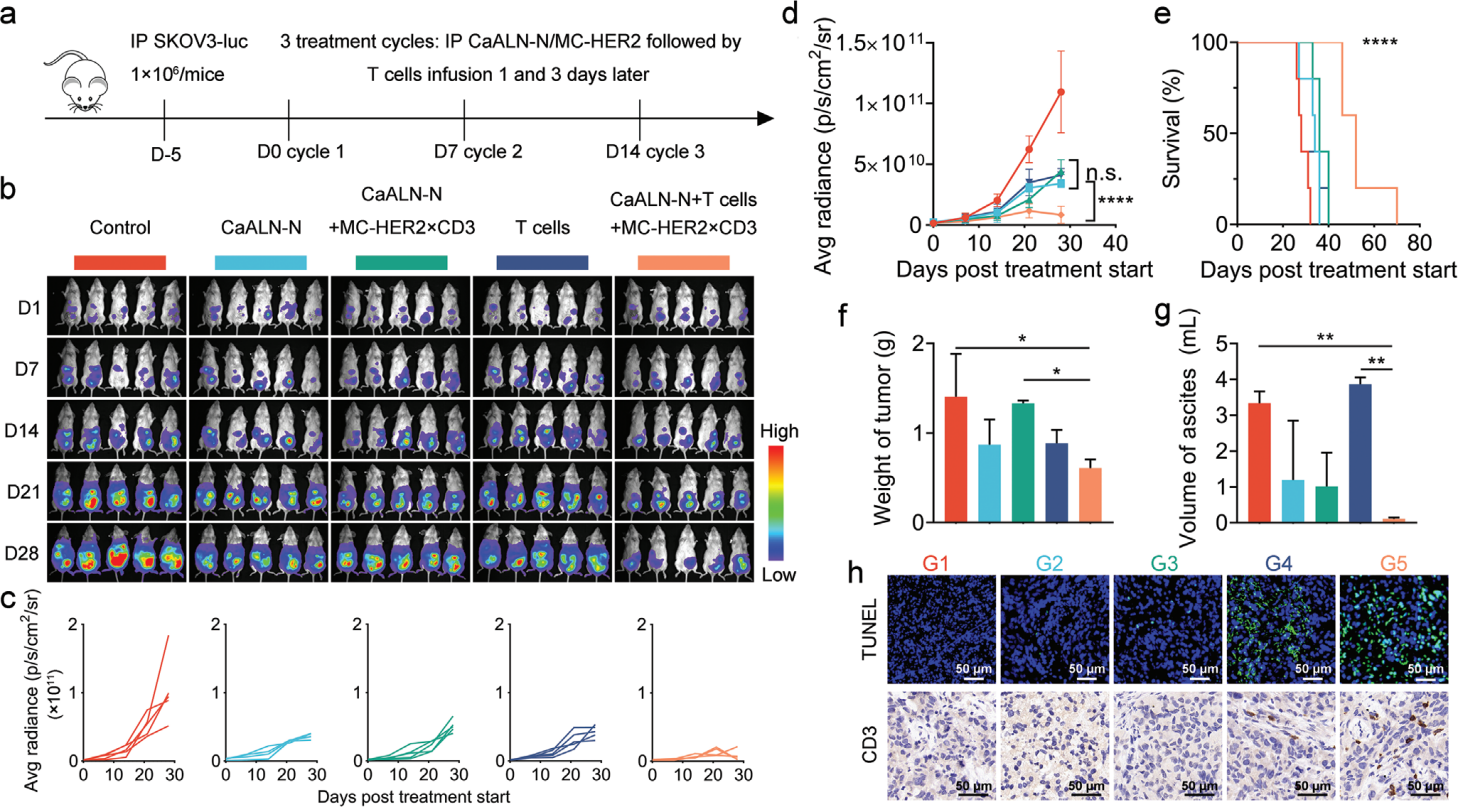
In vivo antitumor effects of CaALN‐N/MC‐HER2×CD3 in human T cells reconstituted human ovarian cancer‐bearing model. a) The timeline of animal experiments. b) In vivo bioluminescence images of mice after different treatments (*n* = 5). c) Average fluorescence intensity of mice in different treatment groups (*n* = 5, mean ± SD). d) Kaplan–Meier survival curves of mice in different treatment groups (*n* = 5, mean ± SD). e) Tumor weight and f) volume of ascites on day 28 (*n* = 5, mean ± SD). g) Histology and immunocytochemical analysis of the tumor. h) Representative images of TUNEL staining and CD3 immunohistochemistry assay. The scale bar was 50 µm.*p < 0.05, **p < 0.01, ****p < 0.0001

BiTEs are Fc‐deficient, which have a relatively short plasma half‐life and suffer from instability and aggregation issues in manufacturing process. Moreover, the clinical application of BiTEs in solid tumor has been hampered presumably by not only the high cost but also the low response rate, partly attributing to systemic toxicity and low concentration in tumor site. The gene delivery potential and synergistic antitumor effects indicated the potential application of CaALN‐N combined with gene therapy for cancer treatment. Next, we further investigated whether CaALN‐N/MC‐HER2×CD3 could generate superior antitumor effect in vivo (**Figure** **6**a).

NOD/SCID mice were grafted with SKOV3‐luc (5 × 10^6^ per mice) via IP injection and tumor growth was monitored using in vivo bioluminescence imaging. Five days after tumor cell injection, tumor‐bearing mice were received IP injection of 100 µg of CaALN‐N and 20 µg of MC‐HER2×CD3. Consist with the expression pattern of luciferase, serum concentration of HER2×CD3 peaked at 24 h at 3.5 ng mL^−1^ and gradually decreased to 0.26 ng mL^−1^ over a 6‐day period (Figure S12, Supporting Information). CaALN‐N could efficiently deliver MC‐HER2×CD3 by IP injection and produce therapeutic level of HER2×CD3 for 1 week, which could overcome the limitations of exogenously administered BiTE using continuous bolus intravenous infusion.

Next, the tumor‐bearing mice received three treatment cycles. One treatment cycle was defined as: IP injection of 400 µL transfection complex containing 100 µg of CaALN‐N with or without 20 µg of MC‐HER2×CD3 on day 0, and adoptive transfer of 1 × 10^7^ ex vivo‐expanded T cells on day 1 and 3 followed by a 4 days‐interval. SKOV3‐luc bearing mice received total three treatment cycles and tumor progression was monitored by IVIS. As shown in Figure 6b–d,f, mice treated with CaALN‐N, CaALN‐N/MC‐HER2×CD3 or adoptive transfer of ex vivo‐expanded T cells alone slightly reduced tumor burden compared to mice of the control group. However, infusion of T cells alone was inefficient to suppress the formation of ascites (Figure 6g). The CaALN‐N/MC‐HER2×CD3 plus T cells dramatically impeded tumor progression and improved the overall survival rate in comparison to other monotherapy (Figure 6e), and very small ascites was collected at the endpoint (Figure 6g). Moreover, no obvious changes in body weight were observed in all treatment groups following three cycles compared with the control group (Figure S13, Supporting Information).

The poor tumor tissue infiltration, immunosuppressive tumor microenvironment (TME), and side effects are prominent drawbacks that hamper the clinical efficacy of T cells‐engaged therapy.^[^ ^7^, ^39^, ^40^ ^]^ Of note, ovarian cancer encompasses dense extracellular matrix and immunosuppressive microenvironment, hindering T‐cell homing, infiltration, and activation.^[^ ^41^ ^]^ To further explore whether TME was reshaped to favor CD3 T cell responses, we analyzed the histopathological features of tumor tissues post the last treatment cycle. Immunostaining of CD3 and terminal‐deoxynucleoitidyl transferase mediated nick end labeling (TUNEL) assay indicated a higher degree of T cell infiltration and increased apoptosis of cancer cells in the CaALN/MC‐HER2×CD3 plus T cells treated group, which confirmed that T cells from peripheral tissue trafficked into tumor and performed their cytolytic effector function (Figure 6h). These data were consistent with in vitro results (Figure 5h–i). The widespread apoptosis of tumor cells indicated the dense structure of tumor was broken and defective to limit T cells infiltration. In addition, the infiltrated T cells were accompanied by apoptotic cancer cells at 14 day post last treatment cycle, which highlighted the importance of redirecting T cells to tumor site for durable antitumor response. Taken together, CaALN‐N considerably improved tumor microenvironment and rejuvenated T‐cell immunity.

In fact, an immunosuppressive outcome follows the presence of metabolic alterations, many types of immune cells (such as myeloid‐derived cells, macrophages, and Tregs) and stroma cells (such as fibroblasts), and their immunoregulatory cytokines.^[^ ^42^, ^43^, ^44^ ^]^ All these components play a role in limiting the activity of T cells recruited from peripheral tissue or pre‐existing in the tumor. In this work, immune reconstitution of immunodeficient mice was generated by infusion of expanded human T cells. However, this short‐term immune reconstitution model lacked syngeneic antigen‐presenting cells (dendritic cells, macrophages, and B cells, etc.) and was unable to accurately simulate physiological immunosuppression microenvironment. CaALN‐N reshaped immunophenotypic signature in syngeneic tumor‐bearing human CD3 transgenic mice and humanized mouse models should also be tested in the future.

**Figure 7:**
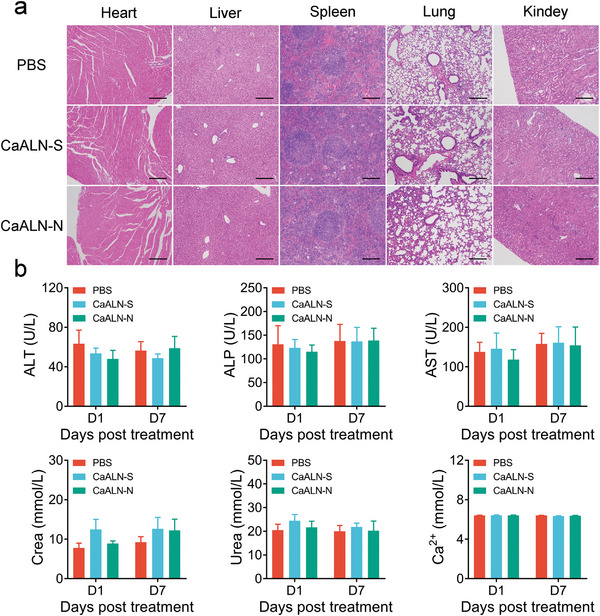
Toxicity assay of CaALN in vivo. 400 µL Ca^2+^/Mg^2+^ free PBS containing 20 µg MC‐luciferase and 200 µg CaALN‐N or CaALN‐S were IP injected into BALB/c mice. a) At the end of the study, mice were sacrificed and major organs were dissected for histopathological analysis. The scale bar was 200 µm. b) The serum samples were collected for AST, ALT, ALP, urea, creatinine, and serum calcium detection (*n* = 5, mean ± SD).

We further evaluated the systemic toxicity of CaALN‐N treatment. There were no macroscopic or microscopic changes in major organs during the terminal necropsy in treated animals at a dose of 10 mg kg^−1^ (**Figure** **7**a). Mice treated with both CaALN‐N and CaALN‐S had no significant change in biochemical analysis, including serum alanine transaminase, aspartate aminotransferase, alkaline phosphatase, creatinine, urea, and blood calcium at 12 h postinjection (Figure 7b), implying good biocompatibility of CaALN biomaterials.

In contrast to the great success in hematological malignancies, the outcome of patients with solid tumors remained suboptimal, underscoring the need for combinatorial approaches to improve the antitumor activity of T‐cell‐based therapeutic agents. Evidence suggested that tailored combination of cancer immunotherapies and nanotechnology were required to achieve optimal therapeutic benefits.^[^ ^45^ ^]^ The ability of chemotherapy and targeted therapies to eliminate the bulk of tumor cells and reshape the tumor microenvironment has been shown in clinical trials of cancer immunotherapies.^[^ ^46^ ^]^ We proposed that the safety and gene‐delivery efficiency of the calcium alendronate nanoparticles together with the synergistic antitumor activity enabled the rational design of multifunctional nanoparticles consisting of adjuvant/chemotherapeutic drugs and optimal combination immunotherapy to treat different cancer types.

## 3 Conclusion

In this present work, an intraperitoneal injected calcium alendronate (CaALN) based gene delivery system was rationally engineered for chemo/immuno‐therapy synergistic treatment of ovarian cancer. The nanoneedle‐like CaALN (CaALN‐N) with a high aspect ratio enabled efficient gene delivery of minicircle encoding HER2×CD3 to the peritoneum without systemic toxicity in vivo and synergized with HER2×CD3 to exhibit superior therapeutic outcomes against human ovarian cancer, which provided a promising strategy for the treatment of abdominal tumors.

## 4 Experimental Section

### Materials

Alendronate sodium trihydrate, CaCl_2_, ethanol, and NaOH were purchased from Sigma‐Aldrich. All chemical reagents were analytically pure and used without further purification. Fetal bovine serum (FBS), phosphate‐buffered saline, and lymphocyte serum‐free medium were purchased from Corning. McCoy's 5A medium, penicillin‐streptomycin (100×), and 0.25% Trypsin‐EDTA were purchased from Cytiva. Calcein‐AM and propidium iodide (PI) were purchased from Dojindo. Human TNF‐*α* and IFN‐*γ* ELISA kits were purchased from Abcam. Firefly luciferase substrate XenoLight D‐luciferin potassium salt was purchased from PerkinElmer. Female BALB/c mice (5 weeks), nude mice (5 weeks), and NOD/SCID mice (5 weeks) were purchased from Beijing Vital River Laboratory Animal Technology Co., Ltd. (Beijing, China). All mice were maintained under specific pathogen‐free conditions. Animal care and experiments were carried out under institutional and National Institutes of Health protocol and guidelines. Animal work described in this manuscript has been approved and conducted under the oversight of the Animal Care and Use Committee of Sun Yat‐sen University (SYSU‐IACUC‐2021‐B0815).

### Cell Lines and Cell Samples

SKOV3 and Human Kidney‐2 (HK2) cells were purchased from the American Type Culture Collection and the luciferase transduced variants of SKOV3 (SKOV3‐luc) were established as described previously.^[^ ^47^ ^]^ The firefly luciferase‐positive NCI‐N87‐luc was purchased from Cobioer Ltd. (Nanjing China). Human peritoneal mesothelial cells (HMrSV5) were gifted by Prof. Zhi‐Feng Miao (First Hospital of China Medical University, Shenyang, China). Cell line identities were confirmed by using DNA‐fingerprinting techniques such as short tandem repeat profiling. All cell lines were checked negative for mycoplasma contamination. After informed consent on Shenzhen Institutes of Advanced Technology, Chinese Academy of Sciences approved protocols (SIAT‐IRB‐170315‐H0146), peripheral blood mononuclear cells (PBMCs) from healthy donors were obtained by Ficoll density centrifugation, activated, and expanded as described previously.^[^ ^47^ ^]^

### Synthesis and Characterization of CaALN

CaALN samples with different morphology and crystal structure were synthesized by orthogonal experiment design. Detailly, alendronate sodium trihydrate solution and CaCl_2_ solution were mixed, and the pH of this solution was adjusted by 0.1 m NaOH. The variable factors of this experiment were P/Ca, pH value, and reaction temperature. At the end of the experiment, the precipitated product was collected, washed twice with ethanol and freeze‐dried. The morphologies of CaALN were obtained with the scanning electron microscope (SEM, FEI Magellan 400, USA) and transmission electron microscope (TEM, JEM‐2100F, Japan). X‐ray diffraction (XRD) was obtained by Rigaku Ultima IV, Japan. Fourier transform infrared spectroscopy (FTIR) spectra were conducted on a Nicolet 7000‐C spectrometer in the range of 400–400 cm^−1^. The thermogravimetric (TG) was recorded on a simultaneous thermal analyzer in flowing air (Netzsch, STA‐409PC, Germany).

### CaALN‐Mediated Gene Delivery In Vivo

Titrated doses of CaALN ranging from 2.5 to 20 mg kg^−1^ and 20 µg MC‐luciferase in 400 µL Ca^2+^/Mg^2+^ free PBS were IP injected into BALB/c mice. PEI 25 K was used as control following recommended instructions. Gene expression level was monitored by firefly luciferase imaging using the IVIS spectrum imaging system (PerkinElmer, USA). The serum samples were collected for AST, ALT, urea, and creatinine detection. To further determine luciferase positive tissue, mice were sacrificed and major organs were dissected 24 h after treatment. Luciferase positive tissue samples were confirmed by bioluminescence imaging.

### Cytotoxicity of CaALN In Vitro

SKOV3‐luc cells, HK2 and HMrSV5 were incubated with various concentrations of CaALN for the indicated time. Cytotoxicity of CaALN was measured by bright‐glo luciferase assay system (for SKOV3‐luc) or CCK8 kit (for HK2 and HMrSV5) following their instructions.

### Migration Assay

Migration was evaluated using scratch wound assay. SKOV3‐luc cells were seeded in a 6‐well plate until confluent monolayer was formed. The scratch wounds were created by scraping cell monolayers with a sterile disposable rubber as previously described.^[^ ^48^ ^]^ CaALN was added into the medium at indicated concentration and further incubated for 24 h. The distance of scratch in the monolayer was quantified and was calculated relative to 0 h.

### RNA‐seq

SKOV3‐luc cells were treated with CaALN‐N (100 mg L^−1^) for 24 h (three biological replicates). Total RNA was extracted using Trizol regent according to the manufacturer protocol. RNA samples were treated with DNase I to remove residual DNA. The total RNA quality and quantity were determined using the Qubit 2.0 (Thermo Fisher Scientific) and the Bioanalyzer 2100 (Agilent Technologies). RNA‐seq libraries were prepared from poly(A)‐enriched mRNA as previously described.^[^ ^49^ ^]^ Libraries were size selected by gel extraction and sequenced on a Hiseq 2500 (Illumina, PE150) by Gene Denovo Biotechnology Co. (Guangzhou, China). The high‐quality clean reads were filtered by fastp and mapped to the human reference genome (GRCh38) using HISAT2. 2.4.^[^ ^50^, ^51^ ^]^ Differential expression analysis was performed with DESeq2 with default parameters.^[^ ^52^ ^]^ Genes with false discovery rate (FDR) below 0.05 and absolute fold change ≥ 2 were considered differentially expressed genes/transcripts.

### Design of HER2×CD3 and Construction of Minicircle DNA Expression Vector

HER2×CD3 was constructed using the classical tandem single‐chain variable fragments (scFv) format. The variable region sequences of anti‐HER2 and anti‐CD3 were from trastuzumab (PDB No. 1N8Z) and solitomab (IMGT/mAb‐DB No. 405) respectively and connected through a flexible linker in the VL_HER2_‐VH_HER2_‐VH_CD3_‐VL_CD3_ orientation. 6×His‐tag and Flag tag sequence were inserted at the N‐ and C‐terminal, respectively. The codon‐optimized constructs were synthesized by synbio‐tech (Suzhou China) and cloned into pMC‐BESXP as a parental plasmid.^[^ ^53^ ^]^ *Escherichia coli* strain ZYCY10P3S2T was transfected with parental plasmid and incubated in 200 mL TB medium at 37 °C with shaking at 250 rpm for 16 h, followed by addition of induction medium (200 mL of LB medium, 200 µL of 20% larabinose, and 8.0 mL of 1 N NaOH) and incubation for 5 h at 32 °C with shaking at 250 rpm. MC‐HER2×CD3 was isolated from bacterial lysates using plasmid purification kits (Qiagen). HER2×CD3 was produced by transient transfection of MC‐HER2×CD3 expression vector into 293F cells, and the supernatant from transfected cells was purified using imidazole‐gradient elution from the Ni‐Sepharose column. The purity of HER2×CD3 was assessed by sodium dodecyl sulfate‐polyacrylamide gel electrophoresis (SDS‐PAGE).

### Binding Assay by Flow Cytometry

To evaluate the binding affinity of HER2×CD3, T cells, and HER2 positive human cancer cells (SKOV3‐luc and NCI‐N87‐luc) cells were suspended in cell staining buffer (BioLegend) and incubated with serially titrated HER2×CD3 for 1 h on ice and then with APC‐conjugated anti‐his antibody for 30 min in the dark. Staining controls were incubated with anti‐his antibody in the absence of HER2×CD3. After washing twice with prechilled PBS, samples were performed on CytoFLEX (Beckman) and analyzed using FlowJo X software.

### HER2×CD3‐Redirected T‐Cell Cytotoxicity

T cells and luciferase transfected target cells were adjusted to 2 × 10^5^ and 2 × 10^4^ mL^−1^, respectively, and 50 µL of each cell suspension was planted into a white 96‐well flat bottom plate at an E/T ratio of 10:1. The HER2×CD3 at various concentrations was added and incubated for 14 h. The cytotoxicity was measured by luciferase‐based bioluminescence assay using a bright‐glo luciferase assay system following their instructions. Furthermore, after 12 h incubation with HER2×CD3 at 1 ng mL^−1^, the cells were stained with calcein‐AM and PI, and photographed using an inverted fluorescence microscope.

### HER2×CD3 Mediated Cytokine Release

The HER2×CD3 at various concentrations was incubated with T cells and SKOV3‐luc at E/T ratio of 10:1 for 16 h. The supernatants of cell culture were harvested and cytokine release was measured by a commercial ELISA kit for IFN‐*γ* and TNF‐*α* (Abcam).

### In Vivo Antitumor Efficacy of CaALN‐N/MC‐HER2×CD3

Female NOD/SCID mice were IP injected with 5 × 10^6^ SKOV3‐luc cells. After 5 days, the tumor engraftment was confirmed by bioluminescence imaging. Before treatment, mice were randomized into 5 groups based on bioluminescence to ensure an evenly distributed average tumor burden across each group. The mice received three treatment cycles: IP injection of CaALN‐N 100 µg with or without 20 µg MC‐HER2×CD3 in 400 µL Ca^2+^/Mg^2+^ free PBS on day 0 and 1 × 10^7^ ex vivo‐expanded T cells on day 1 and 3 or an equal volume of 0.9% saline as a control. A time interval of 4 days was allowed between each treatment cycle. Blood samples were collected on day 1, 3, and 6 for the determination of secreted BsAb‐ HER2×CD3 and cytokines. Tumor growth was monitored by bioluminescent imaging weekly. Some of the animals were sacrificed on day 28 and tumor tissues were excised, fixed in 4% paraformaldehyde buffered saline and embedded in paraffin for TUNEL and immunohistochemistry assay. Formalin‐fixed paraffin‐embedded tissue (5 µm) slides were deparaffinized and stained with a primary anti‐CD3 antibody (ab17143, Abcam) followed by incubation with a HRP conjugated secondary antibody. The slides were visualized using a DAB kit and scanned using Pannoramic MIDI (3DHISTECH).

### Statistical Analysis

All data analyses were performed with GraphPad Prism statistical software (v7.0) and shown as mean ± standard deviation (SD). Statistical comparisons were performed using one‐way analysis of variance (ANOVA) or unpaired two‐tailed *t*‐tests. *p* values of less than 0.05 were considered statistically significant.

## Conflict of Interest

The authors declare no conflict of interest.

## Supporting information

Supporting Information

## Data Availability

The data that support the findings of this study are available on request from the corresponding author. The data are not publicly available due to privacy or ethical restrictions.
