# Supplementary material for: Engineering Bifunctional Calcium Alendronate Gene‐Delivery Nanoneedle for Synergistic Chemo/Immuno‐Therapy Against HER2 Positive Ovarian Cancer
Source: Adv Sci (Weinh). 2023 Mar 18;10(14):2204654. doi: 10.1002/advs.202204654 (PMC10190658; doi:10.1002/advs.202204654)
Supplement: Supplementary file 1 — Supporting Information [file ADVS-10-2204654-s001.pdf]

ADVANCED SCIENCE
Open Access

Supporting Information

for *Adv. Sci.*, DOI 10.1002/advs.202204654

Engineering Bifunctional Calcium Alendronate Gene-Delivery Nanoneedle for Synergistic Chemo/Immuno-Therapy Against HER2 Positive Ovarian Cancer

Guochuang Chen, Leli Zeng, Bo Bi, Xiuyu Huang, Miaojuan Qiu, Ping Chen, Zhi-Ying Chen, Yulong He, Yihang Pan, Yu Chen* and Jing Zhao**

Supplementary Figures

Engineering bifunctional calcium alendronate gene-delivery nanoneedle for synergistic chemo/immuno-therapy against HER2 positive ovarian cancer

Guochuang Chen†, Leli Zeng †, Bo Bi, Xiuyu Huang, Miaojuan Qiu, Ping Chen, Zhiying Chen, Yulong He, Yihang Pan, Yu Chen* and Jing Zhao**

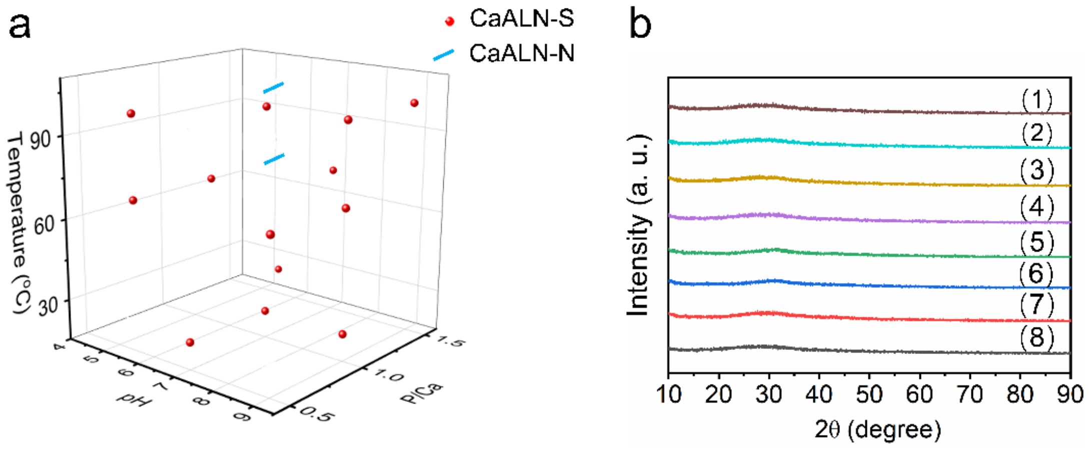

Figure S1. Controllable synthesis of (a) CaALN and (b) representative XRD patterns of CaALN-S. (1) 25 °C, P/Ca of 1 and pH of 9; (2) 25 °C, P/Ca of 1.0 and pH of 7; (3) 25 °C, P/Ca of 0.5 and pH of 7; (4) 25 °C, P/Ca of 1.5 and pH of 5.5; (5) 70 °C, P/Ca of 1.5 and pH of 7; (6) 70 °C, P/Ca of 1 and pH of 7.0; (7) 90 °C, P/Ca of 0.5 and pH of 5; (8) 90 °C, P/Ca of 0.5 and pH of 5.5.

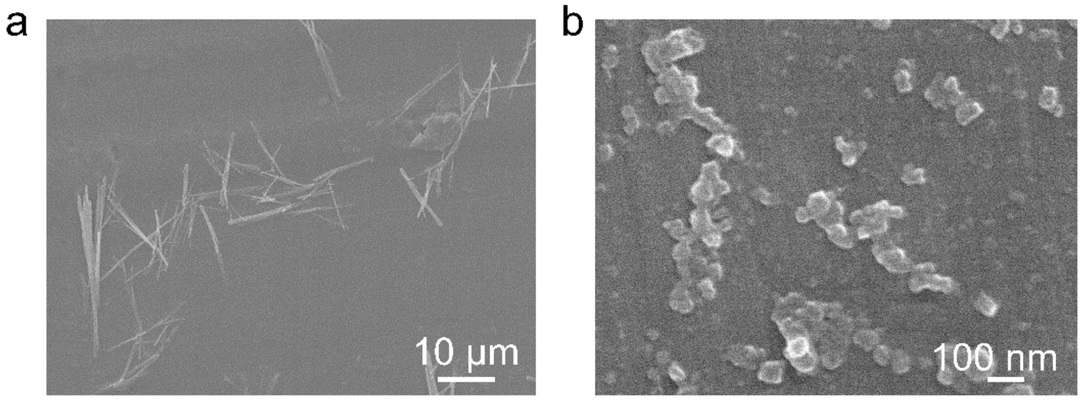

Figure S2. SEM images of a) CaALN-N and b) CaALN-S.

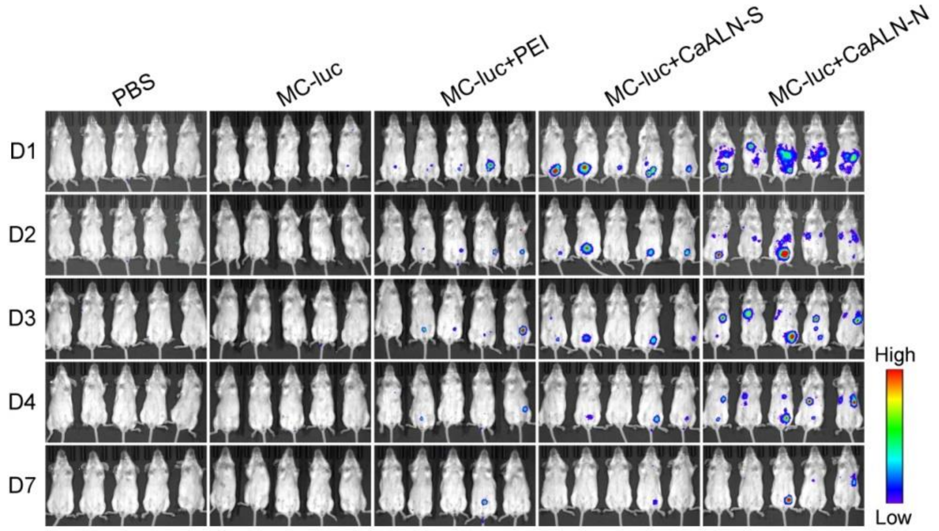

Figure S3. IVIS images of individual mouse corresponding to Fig. 3b.

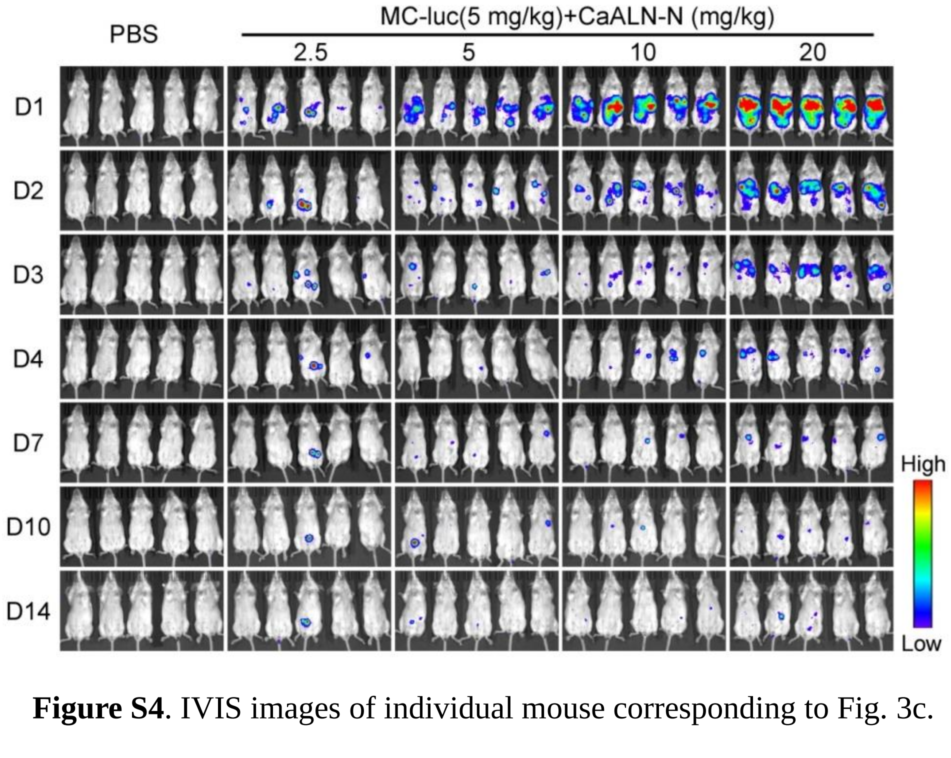

Figure S4. IVIS images of individual mouse corresponding to Fig. 3c.

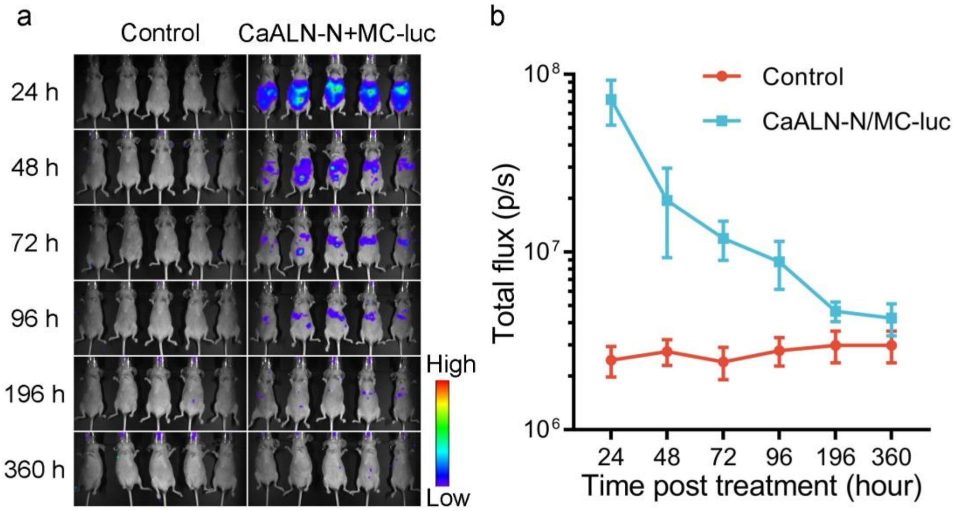

Figure S5. Determination of CaALN-N mediated gene transfection in nude mice. BALB/c nude mice were IP injected with 100 μg CaALN-N and 20 μg MC-luc in 400 μl PBS buffer and the expression of luciferase was monitored by IVIS spectrum imaging system (Tanon, China). a) IVIS images of mice post treatment. b) The transfection efficiency was quantified as bioluminescence signal (total flux) at the indicated time points (n = 5, mean ± SD).

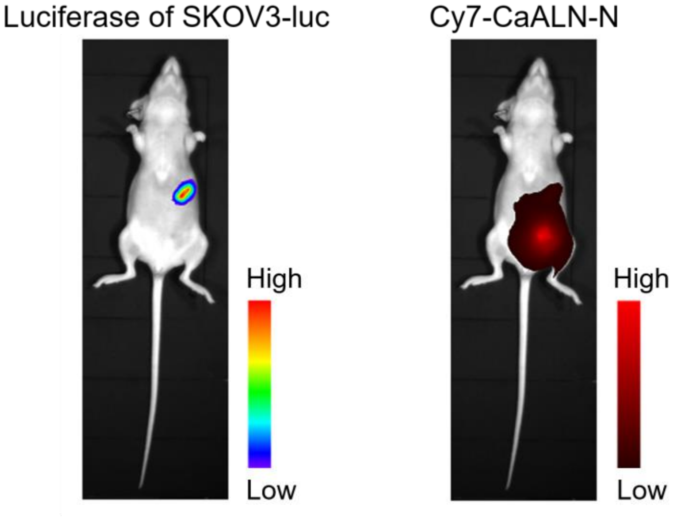

Figure S6. The distribution of CaALN-N *in vivo*. The female nude BALB/c mice were engrafted with SKOV3-luc cells for 5 days. The tumor-bearing mouse was IP injected with Cy7 conjugated CaALN-N (10 mg/kg) and monitored by IVIS spectrum imaging system (Tanon, China). The left: bioluminescence of luciferase; The right: fluorescence (Ex/Em= 780/800 nm) of Cy7.

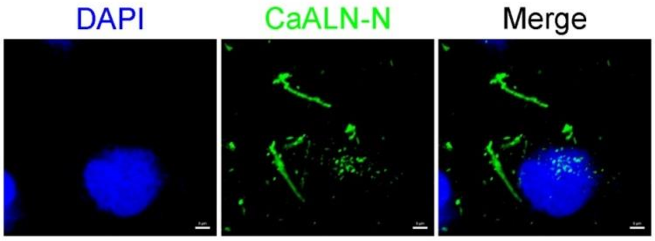

Figure S7. The interaction of CaALN-N and adherent cells *in vitro*. CaALN-N was conjugated with FITC and incubated with SKOV3-luc cells for 24 h. The treated cells were washed twice with PBS and fixed with 4% PFA for 8 min, then stained with DAPI, followed by visualized with confocal microscopy. The scale bar was 5 μm.

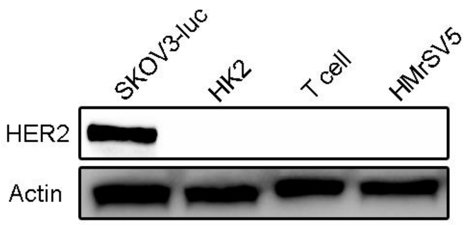

Figure S8. HER2 expression of SKOV3-luc cells, HK2, HMrSV5 and T cells. Total protein was extracted from cell pellets with RIPA buffer (50 mM Tris pH 8.0, 150 mM NaCl, 1% Nonidet P40, 0.5% sodium deoxycholate,0.1% SDS) supplemented with PMSF. Western-blotting was performed with established protocol using mouse monoclonal [3B5] to HER2 (Abcam) and horseradish peroxidase-conjugated goat anti-mouse antibody (Beyotime).

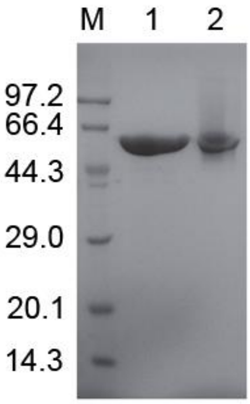

Figure S9. The characterization of HER2×CD3. HER2×CD3 was verified by western-blot under reduced (left) and non-reduced (right) conditions.

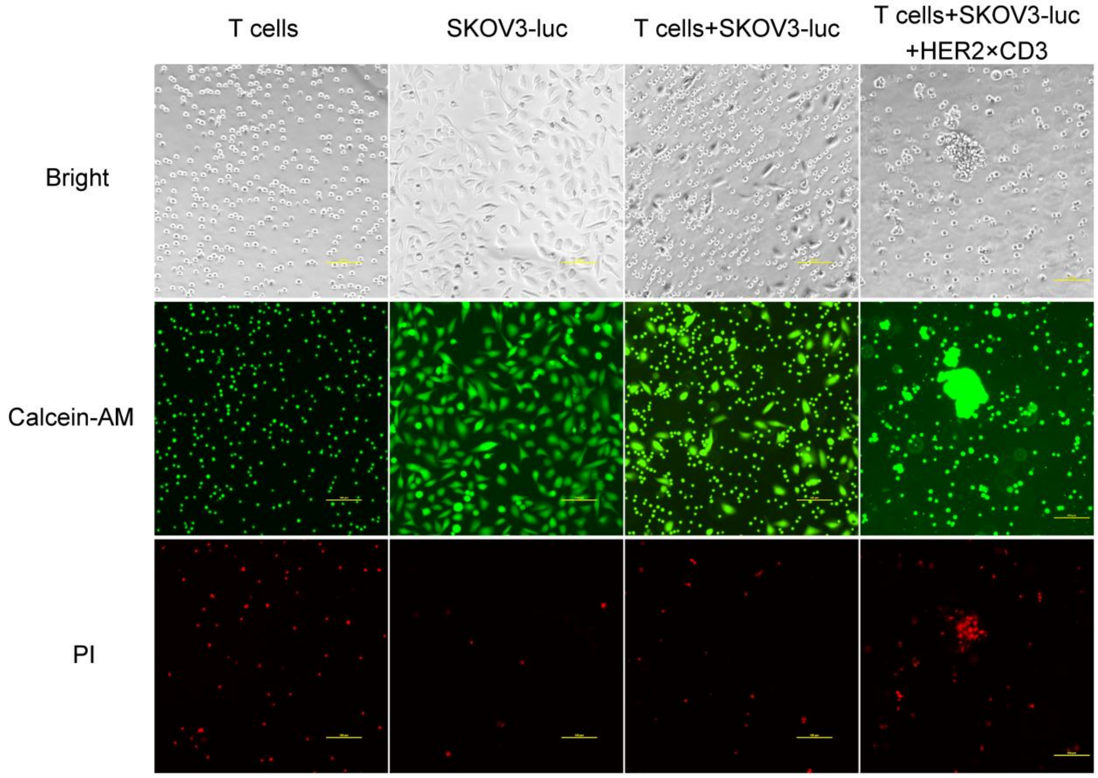

Figure S10. Representative images of cellular morphology by propidium iodide and calcein staining corresponding to Fig. 5d. The scale bar was 100 μm.

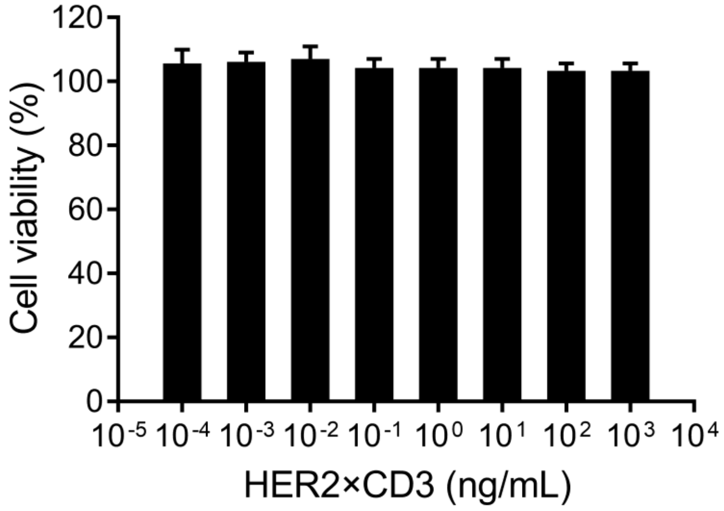

Figure S11. HER2×CD3 showed no direct toxicity on SKOV3-luc cells *in vitro*. SKOV3-luc cells were seeded in 96-well plate and incubated with titrated dose of HER2×CD3 for 24 h, followed by bright-glo luciferase assay (n = 3, mean ± SD).

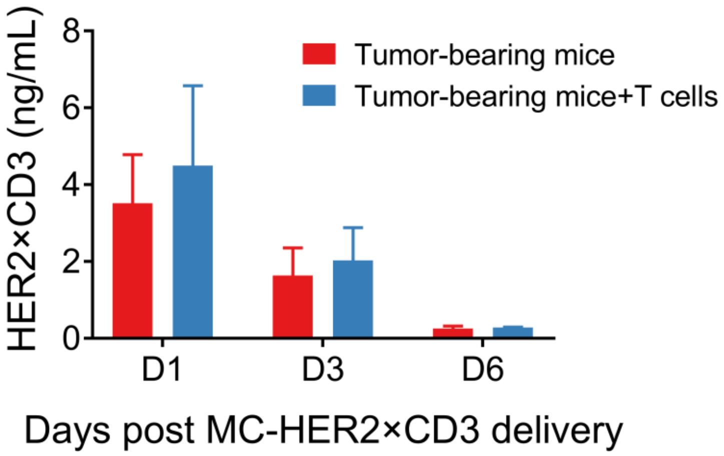

Figure S12. CaALN/MC-HER2 treatment resulted in sustained therapeutic levels of HER2×CD3 *in vivo*. 400 µl transfection complex containing 100 µg CaALN-N with 20 µg MC-HER2×CD3was IP injected on day 0, and adoptive transfer of 1 × 10^7human T cells on day 1 and 3. Mouse peripheral blood was collected from the retro-orbital puncture of the mice at day 1, 3 and 6. The serum levels of HER2×CD3 were measured by T cell directed cellular cytotoxicity assay (n = 4, mean ± SD).

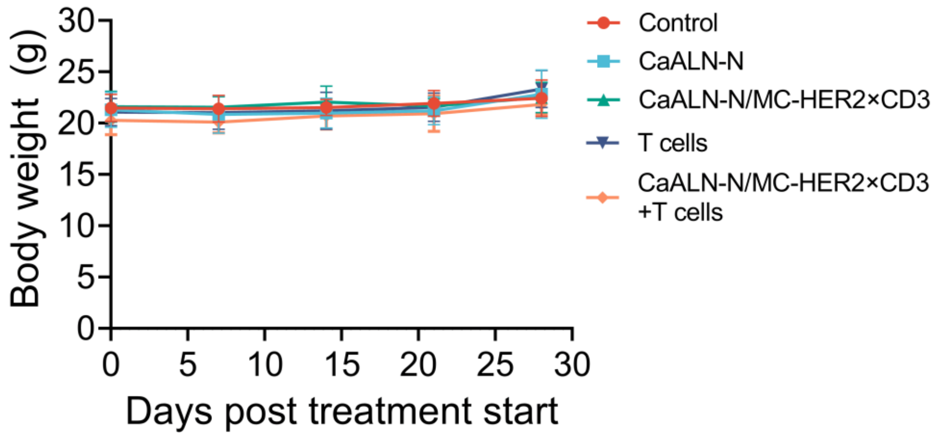

Figure S13. Body weight curves of individual mouse corresponding to Fig. 6b. The body weight was measured at indicated time point (n = 5, mean ± SD).
